# Central retinal vein prethrombosis as an initial manifestation of protein S deficiency

**DOI:** 10.1590/S1516-31802004000300012

**Published:** 2004-05-06

**Authors:** Paulo de Tarso Ponte Pierre, Alessandra Maria Mont'Alverne Pierre, Maurício Abujamra Nascimento, Ana Maria Marcondes

**Keywords:** Retinal vein, Protein S deficiency, Vascular diseases, Case reports, Veia retiniana, Deficiência de proteína S, Doenças vasculares, Relatos de casos

## Abstract

**CONTEXT:**

Retinal vein thrombosis is most common in old people, and is often associated with systemic vascular disease. One of its rare systemic causes is protein S deficiency.

**CASE REPORT:**

A case of a 21-year-old woman with retinal vein prethrombosis associated only with protein S deficiency is described. She presented with acutely reduced visual acuity and a central scotoma in her left eye. Warfarin therapy was initiated, and complete improvement in ophthalmoscopic findings was subsequently observed. This case illustrates that protein S deficiency is a factor that should be considered in cases of retinal vein occlusion, particularly in young patients.

## RESUMO

**CONTEXTO:**

Trombose venosa retiniana é mais comum em pessoas idosas e freqüentemente está associada com doença vascular sistêmica. Uma causa sistêmica rara é a deficiência de proteína S.

**RELATO DE CASO:**

Um caso de paciente branca, de 21 anos, com pré-trombose de veia central da retina associada à deficiência isolada de proteína S é descrito. Ela apresentou súbito embaçamento visual e escotoma central em olho esquerdo. Terapia cumarínica foi iniciada e completa remissão dos achados oftalmoscópicos foi observada. Este caso mostra que deficiência de proteína S deve ser um fator que deve ser considerado em casos de oclusão venosa retiniana, particularmente em pacientes jovens.

## INTRODUCTION

Central retinal vein thrombosis is typically a disease of older patients, rarely occurring in young adults. It is often associated with either an underlying vascular disease or a procoagulant state.^1^ Hypertension, diabetes, vasculitides, and dyslipidemia are the most commonly associated vascular disorders, while the recognized procoagulant states include thrombocytosis, polycythemia, macroglobulinemia, the use of oral contraceptive pill or systemic lupus erythematosus.

Protein S and protein C are vitamin K-dependent plasma proteins that modulate coagulation. Protein S serves as a cofactor for protein C to inhibit the clotting cascade at the levels of factors V and VIII.^2^ There are few reports of retinal vein thrombosis associated with protein S deficiency,^3,4^ and these have usually occurred in combination with other thrombophilic conditions. This case report describes a young patient with protein S deficiency who developed central retinal vein prethrombosis. No other prothrombotic risk factors were present.

## CASE REPORT

**Figure 1:**
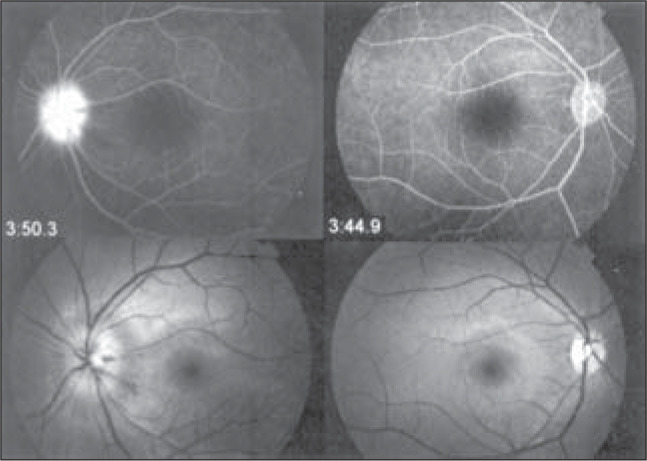
Bottom: Photograph showing rare dot and blot hemorrhages and blurring of optic disc in the left eye. The optic disc, macula, and retinal vessels appear normal in the right eye. Top: Fluorescein angiogram showing late optic disc hyperfluorescence; the disc is extremely edematous on the left.

A 21-year-old white woman presented with acutely reduced visual acuity and a central scotoma in her left eye. She did not present photophobia, ocular pain, fever or any other local or systemic complaint. The patient had no personal or family history of eye disease, systemic hypertension, diabetes mellitus, thrombophilia, malignancy or use of oral contraceptive pills. Her best-corrected visual acuity was 20/15 in the right eye and 20/25 in the left. The anterior segment examination was unremarkable, without papillary defects, and the intraocular pressures were 12 mmHg in the right eye and 11 mmHg in the left eye. Ophthalmoscopy of the left eye showed rare dot and blot hemorrhages and blurring of the optic disc. The retinal venules appeared moderately tortuous but undilated. In the right eye, the ophthalmoscopy was normal. Fluorescein angiography of the left eye revealed normal arterial filling but delayed arteriovenous filling, and there was late disc hyperfluorescence compatible with prethrombosis (Figure 1). However, the right eye was normal. The results from hematological tests showed an erythrocyte sedimentation rate of 18 mm/hour, and the complete blood count, prothrombin time, partial thromboplastin time and anticardiolipin antibodies were normal. She was negative for anti-nuclear antibodies, rheumatoid factor, lupus anticoagulant antiphospholipid antibodies, and serum protein electrophoresis. The activated protein C resistance, plasma levels of protein C and antithrombin III were normal. Decreased plasma levels were found for both free protein S (6%; normal is 20-40%), and total protein S (36%; normal is 60-140%).

Warfarin therapy was initiated. One month later, the visual acuity of the left eye was 20/20 and complete improvement in ophthalmoscopic findings was noted. Neither parents nor siblings were available for ocular and systemic examination.

## DISCUSSION

Central retinal vein thrombosis in young adults is considered uncommon and has been difficult to characterize because the cause is often unknown. Although there is no consensus, it seems appropriate to refer patients with central retinal vein occlusion for thrombophilia screening if they are younger than 50 years old.^5-7^

Prince et al.^3^ reported a patient with systemic lupus erythematosus and acquired protein S deficiency causing central retinal vein thrombosis. Bertram et al.^7^ measured protein C, protein S, and antithrombin III concentrations in ocular vascular occlusive disease, and found a pathologically reduced anti-thrombin III level in one patient with branch venous occlusion, and severely altered protein C and protein S concentrations in two patients with central retinal vein occlusion.

Protein S deficiency, a condition that may be either inherited or acquired, may result in a hypercoagulable state and it predisposes affected individuals to thrombotic events. In the inherited form, clinical manifestations usually occur before the age of 30 years. It is transmitted as an autosomal dominant trait. The deficiency can be acquired under conditions like septic shock, disseminated intravascular coagulation, acute respiratory distress syndrome, postoperative states, liver diseases, pregnancy, oral contraceptive pill use, human immunodeficiency virus infection and chemo-therapy for breast cancer.^8^ The initial presentation of prethrombosis in this patient was related to protein S deficiency.

Recognition of an association of protein S with thrombotic disease may suggest specific therapeutic approaches. In our patient, therapy with oral anticoagulant was initiated because of the known risk of the recurrence of thrombosis in cases of protein S deficiency.^8^

We suggest that protein S deficiency should be considered in the cases of some patients with unexplained retinal vascular occlusions, particularly when occurring in young patients or when there is a positive family history of thrombotic disease.
